# Barriers and enablers to sustainable anaesthetic practice: a mixed-methods study

**DOI:** 10.1016/j.bja.2025.11.009

**Published:** 2025-12-17

**Authors:** Carys Batcup, Aws Almukhtar, Aarya Menon, Daniel Leff, Gaby Judah, Pelin Demirel, Talya Porat

**Affiliations:** 1 Dyson School of Design Engineering, Imperial College London, London, UK; 2 Faculty of Social and Behavioural Sciences, University of Amsterdam, Amsterdam, The Netherlands; 3 Department of General Surgery, Imperial College Healthcare NHS Trust, St Mary’s Hospital, London, UK; 4 Department of Surgery and Cancer, Imperial College Healthcare NHS Trust, St Mary’s Hospital, London, UK

**Keywords:** behavioural determinants, clinical decision making, general anaesthesia, local anaesthesia, mixed-methods research, operating theatre efficiency, sustainable practice, TIVA

## Abstract

**Background:**

Anaesthetic practices contribute significantly to the environmental impact of healthcare. Using local or regional anaesthesia instead of general anaesthesia, and TIVA instead of inhalation anaesthesia, can reduce this impact. This study investigated why general anaesthesia is sometimes used over local and regional anaesthesia, and why inhalation agents are often chosen over TIVA.

**Methods:**

We conducted a mixed-methods study in the UK (June 2023–April 2024), underpinned by the Theoretical Domains Framework. Semi-structured interviews (*n*=19) with anaesthetists, surgeons, and nurses of differing seniority were analysed using Framework Analysis. A national survey (*n*=347), distributed via posters and professional networks, was developed from early interview findings. Quantitative data were analysed descriptively and open-text responses were coded using the qualitative framework.

**Results:**

Four key themes were identified: (1) contextual factors affecting anaesthesia decision making; (2) patient differences and preferences; (3) influence of key decision makers on anaesthesia choice; and (4) default practices and lack of confidence in alternatives. These encompassed 17 subthemes and mapped to 9 of 14 Theoretical Domains Framework domains.

**Conclusions:**

This study provides new insights into behavioural influences underlying anaesthetic practice, which can inform the design of interventions to improve the sustainability of anaesthesia, without compromising patient safety and comfort. Addressing systemic and behavioural barriers through dedicated local anaesthesia operating lists, improved patient communication, targeted training, and supportive technologies may enhance efficiency while promoting safe, sustainable, patient-centred practice. Future interventions should be co-designed with surgeons, anaesthetists, and patients to ensure clinical acceptability, feasibility, and sustainability.

**Editor’s key points**

• Surgery and anaesthesia have a harmful environmental footprint, which is forecast to grow as surgical numbers increase in coming decades. Part of this footprint is driven by anaesthetists’ choices about the type of anaesthesia and the specific anaesthetic agents to be used.
• In this UK-based study, operating theatre staff were interviewed in a structured manner about anaesthesia practices. Informed by these initial findings, a national survey of operating theatre staff was then conducted.
• Where anaesthetic choice is possible, contextual and logistical constraints strongly influence decisions about general anaesthesia (GA) *vs* local anaesthesia/regional anaesthesia (LA/RA) and inhalation *vs* TIVA GA. Patient comorbidities and preferences were also seen as factors in anaesthesia selection. Decisions about GA *vs* LA/RA were often shaped by professional hierarchies. Entrenched practice patterns, underwritten by the confidence engendered by repetition, were highly influential. Environmental motivations were often overridden by practical considerations.
• This research shows that promoting more sustainable anaesthesia practices will require both systemic and behavioural barriers to be addressed.

Healthcare is a significant contributor to climate change, prompting organisations such as the World Health Organization to prioritise reducing the sector’s environmental impact.^1^^,^^2^ Within healthcare, operating theatres are among the highest sources of carbon emissions,^3^, ^4^, ^5^, ^6^ driven by surgical waste, high energy consumption, and anaesthetic practices.^7^, ^8^, ^9^, ^10^ The environmental impact of anaesthesia is compounded by rising global demand for anaesthesia driven by an ageing population, increasing chronic disease burden, and the growing number of surgical procedures, particularly in outpatient and minimally invasive settings.^11^^,^^12^ This anticipated growth underscores the need to develop sustainable approaches to anaesthetic practice.

Several factors influence the environmental footprint of anaesthetic practice. Inhaled agents such as desflurane and nitrous oxide have particularly high global-warming potential and can markedly increase emissions.^7^^,^^13^^,^^14^ When these agents are avoided, and techniques that are less harmful to the environment are used (e.g. low-flow or TIVA techniques, or local anaesthesia/regional anaesthesia [LA/RA]),^13^^,^^15^, ^16^, ^17^, ^18^, ^19^, ^20^, ^21^ the relative impact of surgical processes and engineering systems, such as equipment sterilisation, material use, and theatre ventilation, becomes more dominant in the overall footprint.^22^, ^23^, ^24^ Even within anaesthesia, additional factors such as oxygen flow rates during LA/RA techniques can significantly influence emissions.^24^^,^^25^

Understanding what factors influence anaesthetic choices is key to promoting sustainable practice. Previous studies have identified barriers to sustainable anaesthetic practices, including limited knowledge and awareness, time constraints, and limited resources.^26^^,^^27^ However, there is very limited research on the determinants of anaesthesia choice, especially in decisions between LA/RA and general anaesthesia (GA) using a behavioural science lens. There is also very limited qualitative research, which adds nuance and depth, especially into unexplored areas.^9^ For example, there are only a few qualitative studies investigating use of LA/RA rather than GA, and these mainly investigated low-income countries or specific types of surgeries (e.g. open inguinal hernia repair).^27^^,^^28^

The aim of this study, therefore, was to investigate the determinants of choosing LA/RA instead of GA and TIVA instead of inhalation agents for GA, and the prevalence of these determinants across the UK. Given the complexity of anaesthesia decisions, encompassing staff roles, patient factors, and organisational influences, and the limited existing work in the area, we planned a mixed-methods approach,^29^^,^^30^ including qualitative interviews and a national survey.

## Methods

This study used a sequential mixed-methods design, comprising semi-structured interviews followed by a national survey. Ethical approval was obtained from the Health Research Authority (HRA) and Health and Care Research Wales (HCRW), reference 23/HRA/0920 (March 23, 2023).

### Interviews

#### Participants and design

Interviews were conducted between June and September 2023. Participants were operating theatre staff (anaesthetists, nurses, operating department practitioners [ODPs], and surgeons) from hospitals within Imperial College Healthcare, a National Health Service (NHS) Trust in London, UK, recruited via posters, staff mailing lists, and professional networks. We used purposive sampling to ensure a mix of professional roles and seniority levels. Predefined sampling targets were five or more surgeons, five or more anaesthetists, five or more nurses (including at least one anaesthetic nurse), and one or more ODP, to capture diverse perspectives on anaesthetic decision making and perioperative practice. Interviews were conducted online or in-person, based on participant preference.

A topic guide informed by the Theoretical Domains Framework (TDF) was used (Supplementary material 1). Interviews began with an open question about the participant’s role, followed by TDF-informed questions about anaesthesia practice. TIVA-specific questions were confined to anaesthetists.

Two researchers conducted the interviews: CB (female, MSc, behavioural scientist with qualitative and sustainability expertise) and AA (male, MBBS, MSc, MRCSEd, MFSTEd, practicing surgeon with sustainability expertise). AA had previous professional acquaintance with a small number of participants; no other previous relationships existed. Participants were informed that the study examined barriers and facilitators to green surgery practices and that interviewers were researchers from Imperial College London. Interviews were audio-recorded, transcribed, and anonymised.

#### Analysis

Analysis was conducted using Framework Analysis,^31^ guided by the TDF.^32^^,^^33^ The TDF synthesises 14 domains from 33 theories of behaviour change, providing a comprehensive and structured approach to examine the determinants of behaviour. In addition to knowledge, beliefs, and skills, it encompasses social influences and environmental context to provide a broad understanding of modifiable factors underlying clinical behaviours. The TDF has been widely used in healthcare, including sustainability research,^9^^,^^34^^,^^35^ and can be used in conjunction with other behavioural frameworks^36^ to identify effective behaviour change techniques for intervention design.

Two researchers (CB and AM) independently coded two transcripts and discussed emerging themes. They then coded additional transcripts, with new themes reviewed collaboratively. The wider team (AA, GJ, PD, TP) contributed to developing a coding framework, which CB applied to the remaining transcripts. Outlying data were discussed with the team to finalise the framework. Subthemes were then deductively mapped to the relevant TDF domains.

### Survey

#### Participants and design

A national survey was conducted via Qualtrics (July 2023–April 2024). Eligible participants were aged 18 yr or above, English-speaking, and currently working in a UK operating theatre. Recruitment included posters, newsletters, and dissemination through professional bodies (e.g. Royal Colleges of Surgeons, Anaesthetists, and Nursing). A £100 prize draw entry was offered as an incentive.

#### Measures

Survey items were based on TDF domains and informed by interview findings. Tailored questions were presented to surgeons, anaesthetists, and nurses. Surgeons and anaesthetists answered questions about the use of LA/RA *vs* GA and whether more procedures could be performed under LA/RA. Although anaesthetists are not typically present for procedures performed solely under LA in the UK, their views were included to capture perceptions of workflow efficiency and relative time demands compared with GA or RA techniques. Nurses indicated their preferences. All participants indicated perceived advantages of each anaesthesia type.

Anaesthetists also answered TIVA-specific questions, including frequency of use and reasons for choice. Free-text questions invited additional comments about LA/RA *vs* GA (all participants) and TIVA *vs* inhalation agents (anaesthetists only). Full survey content is provided in Supplementary material 2.

#### Analysis

Internal consistency for TDF-based items was assessed using Cronbach’s α. When α >0.6, items were averaged to create composite scores. Descriptive statistics were calculated for each item or construct, reported by professional group. Quantitative results are presented as mean (sd). Free-text responses were analysed using the qualitative coding framework developed from the interviews, with new themes identified as needed.

## Results

Findings from the interviews and survey are presented using an integrated thematic approach. Four overarching themes from the qualitative analysis provide a structure for the results, with data from both methods mapped onto each theme. This approach provides a comprehensive understanding of behavioural influences on anaesthetic practice by combining in-depth qualitative insights with quantitative data that complement and expand emerging themes across a broader cohort. This is consistent with best practices in mixed-methods research, where qualitative and quantitative data are analysed independently but integrated during interpretation.^37^

### Participant characteristics

Nineteen participants were interviewed, representing a range of clinical roles and levels of seniority (Table 1). No participants declined participation or withdrew during the study. Thematic saturation was reached after 15 interviews, including after the third anaesthetist interview, with the remaining four interviews confirming saturation across roles. The mean interview duration was 41 min (range, 22–64 min).

**Table 1:** Interview participant characteristics (*n*=19). ODP, operating department practitioner; SHO, senior house officer.

Characteristic	*n* (%)
Sex	
Male	9 (47)
Female	10 (53)
Role in operating theatres	
Nurse	7 (37)
Junior	2 (11)
Senior/theatre manager	4 (21)
ODP	1 (5)
Surgeon	8 (42)
Junior (SHO or below)	2 (11)
Intermediate (registrar)	3 (16)
Senior (consultant)	3 (16)
Anaesthetist	4 (21)
Senior (consultant)	4 (21)

Of 494 individuals who accessed the survey, 347 (70%) met eligibility criteria and completed the survey (Table 2) . Respondents included 110 (32%) surgical nurses and operating theatre practitioners, 72 (21%) surgeons, 118 (34%) anaesthetists, and 47 (14%) ODPs and anaesthesia associates. The majority (315, 91%) were based in England, with 195 (62%) of those in London. Free-text responses were provided by 145 (42%) respondents: 24 surgical nurses, 23 ODPs, 25 surgeons, and 73 anaesthetists.

**Table 2:** Survey participant characteristics (*n*=347). Sex/gender reflects participants’ self-identified gender as reported during the survey.

Variable	*n* (%)
Participant type	
A nurse or operating theatre practitioner (including assistants)	110 (32)
An operating department practitioner or anaesthesia associate	47 (14)
A surgeon	72 (21)
An anaesthetist	118 (34)
Age group (yr)	
18–29	32 (9)
30–39	153 (44)
40–49	94 (27)
50–59	51 (15)
60+	17 (5)
Sex/gender	
Female	190 (55)
Male	153 (44)
Prefer to describe myself as	1 (0.3)
Prefer not to say	3 (1)
Ethnicity	
Asian/Asian British	53 (15)
Black/African/Caribbean/Black British	14 (4)
Mixed/multiple ethnic background	10 (3)
White	249 (72)
Any other ethnic group, please describe	15 (4)
Prefer not to say	6 (2)
Hospital type	
Tertiary hospital	235 (68)
District General Hospital	102 (29)
Rehabilitation, community, or ‘cottage’ hospital	4 (1)
Private hospital	18 (5)
Other	10 (3)
Experience in operating theatres	
<1 yr	11 (3)
1–5 yr	110 (32)
6–10 yr	73 (21)
11–20 yr	73 (21)
>20 yr	80 (23)
Country	
Northern Ireland	1 (0.3)
Wales	15 (4)
Scotland	16 (5)
England	315 (91)
Trained in the UK	
Yes	281 (81)
No	66 (19)

### Integrated themes

Framework analysis identified four themes and 17 subthemes, mapped onto eight of the 14 TDF domains: emotion; environmental context and resources; beliefs about consequences; memory, attention, and decision processes; social influences; goals; beliefs about capabilities; and skills. The themes were: (1) contextual factors affecting anaesthesia decision making; (2) patient differences and preferences; (3) influence of key decision makers on anaesthesia choice; and (4) defaults practices and lack of confidence in alternatives. These are detailed below and in Table 3. Quantitative results from the survey are summarised below and in Table 4, Table 5, Table 6.

**Table 3:** Summary of themes from interviews and survey comments. GA, general anaesthesia; LA, local anaesthesia; RA, regional anaesthesia; TDF, Theoretical Domains Framework.

Themes	Sub themes	TDF domain	Barrier or facilitator
1. Contextual factors affecting anaesthesia decision making	Some specialties or operations do not have options for different anaesthesia types	Environmental context and resources	Barrier
	Funds to support LA/TIVA	Environmental context and resources	Barrier or facilitator
	Time and efficiency	Goals	Barrier
	Sustainability is not viewed as a valid reason for changing anaesthesia practice	Beliefs about consequences	Barrier
	Changes are already underway in anaesthetic practice	Environmental context and resources	Facilitator
2. Patient differences and preferences	Patient comorbidities necessitating particular anaesthesia type	Environmental context and resources	Barrier
	Patient comfort influences anaesthesia choice	Environmental context and resources	Barrier or facilitator
		Social influences	
	Different patient outcomes between GA and LA/RA	Beliefs about consequences	Barrier or facilitator
	Patient preferences are paramount	Social influences	Barrier or facilitator
	Increasing anaesthesia options through addressing patient needs	Goals	Barrier or facilitator
3. Influence of key decision makers on anaesthesia choice	Preferences for GA over LA/RA for non-clinical reasons	Memory, attention, and decision processes	Barrier
	Avoiding discussing different anaesthesia types	Emotion	Barrier
	Differing opinions as to whether LA/RA/TIVA are more sustainable than alternatives	Memory, attention, and decision processes	Barrier or facilitator
		Beliefs about consequences	
	Key decision makers (e.g. surgeons and anaesthetists) strongly influence anaesthesia choices, enabling or limiting sustainable practices	Social influences	Barrier or facilitator
4. Default practices and lack of confidence in alternatives	Conforming to default anaesthesia type	Memory, attention, and decision processes	Barrier or facilitator
	Default anaesthesia differs between contexts	Environmental context and resources	Barrier or Facilitator
	Lack of experience leading to less confidence with alternatives	Skills	Barrier
		Beliefs about capabilities

**Table 4:** Contextual, situational, and practical factors influencing anaesthetic choice. Responses are presented as mean (sd) of a 5-point Likert scale score where 1=never select general anaesthesia and 5=always select general anaesthesia.

	Anaesthetists	Surgeons
Patient preference	4.1 (0.8)	3.7 (0.8)
Anxiety levels of patient	3.7 (0.8)	3.5 (0.7)
Confidence with administering regional block required	3.4 (1.2)	–
Language translation of communication to patient if they are awake	3.2 (1.0)	2.7 (1.1)
Time taken to perform surgery	3.1 (1.1)	2.5 (1.2)
Likelihood of patient movement	3.0 (1.0)	2.6 (0.9)
The number of surgeries you have that day	2.7 (1.1)	2.3 (1.0)
Time of day of the operation	2.5 (1.0)	2.2 (1.0)

**Table 5:** Binary barriers and facilitators to LA and GA (selected as multiple choice questions). Data are given as *n* (%). GA, general anaesthesia; LA, local anaesthesia; TDF, Theoretical Domains Framework.

	TDF or other domain	Nurses	Anaesthesia nurses	Surgeons	Anaesthetists
Benefits of local anaesthesia					
The surgery takes less time	Environmental context and resources	63 (68)	47 (73)	14 (19)	18 (15)
I can talk to the patient	Social influences	48 (52)	31 (48)	23 (32)	35 (30)
	Memory, attention, and decision processes				
The surgery is easier (relative to general anaesthesia)	Beliefs about capabilities	22 (24)	17 (27)	8 (11)	7 (6)
	Beliefs about consequences				
	Skills				
The patient can leave shortly after the surgery	Environmental context and resources	52 (56)	53 (83)	52 (72)	98 (83)
Less patient monitoring required	Environmental context and resources	30 (32)	20 (31)	28 (39)	35 (30)
	Memory, attention, and decision processes				
It is safer for the patient	Beliefs about consequences	37 (40)	42 (66)	35 (49)	61 (52)
It's better for the environment	Beliefs about consequences	32 (34)	38 (59)	34 (47)	81 (69)
	Memory, attention, and decision processes				
None of the above	-	0	1 (2)	1 (1)	8 (7)
Benefits of general anaesthesia					
No concerns about communicating with the trainer or trainee	Social influences	43 (46)	11 (17)	26 (36)	40 (34)
No concerns about discussions within the team that would be inappropriate or distressing for the patient to hear	Social influences	38 (41)	27 (42)	28 (39)	67 (57)
The surgery is easier (relative to local/regional anaesthesia)	Beliefs about capabilities	18 (19)	19 (30)	25 (35)	43 (36)
	Beliefs about consequences				
	Skills				
I don’t have to worry about the patient feeling pain	Beliefs about consequences	42 (45)	31 (48)	40 (56)	52 (44)
	Emotion				
No concerns about the patient feeling anxious	Emotion	42 (45)	37 (58)	41 (57)	86 (73)
I don't have to worry about the patient moving during the operation	Memory, attention, and decision processes	41 (44)	37 (58)	39 (54)	75 (64)
The surgery takes less time	Environmental context and resources	5 (5)	14 (22)	15 (21)	24 (20)
None of the above	-	4 (4)	11 (17)	1 (1)	5 (4)

**Table 6:** Continuous barriers and facilitators for using local, regional, or general anaesthesia. Responses are presented as mean (sd) of a 5-point Likert scale score where 1=strongly disagree and 5=strongly agree. TDF, Theoretical Domains Framework.

Agree–disagree barriers	TDF or other domain	Surgeons	Anaesthetists	Nurses	Anaesthesia nurses
I always present the patient with a regional or local anaesthetic option as well as a general anaesthetic option when clinically appropriate (not including supplementary regional anaesthesia).	-	3.9 (1.1)	4.2 (1.1)		
When deciding on which anaesthesia to use (general, local, or regional), I consider the environmental impact.	Memory, attention, and decision processes	2.7 (1.5)	3.3 (1.5)		
I feel just as confident using local anaesthesia only as I do in a surgery where the patient is under general anaesthesia.	Beliefs about capabilities	3.6 (1.3)			
I feel confident providing epidural/spinal anaesthesia.	Beliefs about capabilities		4.8 (0.6)		
I feel confident providing other regional anaesthesia as needed for my practice.	Beliefs about capabilities		3.8 (1.3)		
I feel confident providing sedation to a patient under local anaesthesia.	Beliefs about capabilities		4.7(0.6)		
Operations under local anaesthesia take me longer than operations under general anaesthesia.	Environmental context and resources	2.4 (1.5)			
It takes me longer to anaesthetise using regional anaesthesia than it does general.	Environmental context and resources Skills		3.7 (1.3)		
We should always aim to do operations under local/regional anaesthesia, if possible.	Goals Intentions	3.9 (1.1)	3.5 (1.4)		
I prefer to perform operations under/use general anaesthesia instead of local/regional anaesthesia.	Decision making	2.8 (1.3)	2.9 (1.3)		
I feel more relaxed in an operation which is performed under general anaesthesia/anaesthetising using general anaesthesia.	Emotion	3.8 (1.2)	3.6 (1.3)		
I feel apprehensive when I have to perform an operation using local anaesthesia/anaesthetising using regional anaesthesia.	Emotion	2.7 (1.34)	2.7 (1.4)		
Some anaesthetists I work with are not as confident doing regional anaesthesia as they are doing general.	Environmental context and resources	4.0 (1.2)	4.6 (0.7)		
Having a dedicated local/regional anaesthesia operating list is feasible and efficient.	Environmental context and resources	4.0 (1.0)	–		
Using general anaesthesia is the default option.	Behavioural regulation	3.4 (1.2)	3.4 (1.2)		
Patients are presented with a choice of local versus general anaesthesia (when possible).	Social influences	3.0 (1.1)	3.8 (1.1)		
My patients tend to prefer general anaesthesia if given the choice.	Social influences	3.3 (1.2)	3.9 (0.9)		
My patients tend to prefer local anaesthesia if given the choice.	Social influences	2.8 (0.9)	2.5 (0.93)		
It is likely that the use of general anaesthesia can be reduced in my hospital.	Optimism	4.1 (1.0)	3.8 (1.1)		
The surgical team I am working with is open to using local/regional anaesthesia instead of general for an operation which could use either. The hospital I work in would support efforts to reduce the number of operations done under general anaesthesia.	Social influences	3.6 (0.9)	3.4 (0.9)		
Reducing the number of surgeries using general anaesthesia would have a positive impact on the environment/on patient care.	Beliefs about consequences	4.1 (1.0)	3.9 (1.2)	3.5 (0.9)	3.6 (1.3)
I believe my Trust is competent/committed at protecting me/patients from infections.	Social influences	3.6 (0.8)	3.6 (0.8)	3.7 (0.7)	3.8 (0.7)
I believe the operating theatre needs to become more environmentally friendly.	Goals	4.6 (0.6)	4.7 (0.7)	4.3 (0.6)	4.4 (1.0)

#### Theme 1—Contextual factors affecting anaesthesia decision making

Participants agreed that GA is essential for many procedures. However, where anaesthetic choice is possible, contextual constraints strongly influence decisions. A recurring issue was lack of institutional prioritisation and funding for increasing LA/RA and TIVA use, resulting in a lack of infrastructure and supplies. Survey participants noted that logistical issues within their facilities reduced the feasibility of RA, as one anaesthetist noted:

*‘Logistics*—*better to have a room where patients could be blocked and wait for high turnover.’* (Consultant anaesthetist, survey)

Others highlighted that hospital management provided these facilities in the right circumstances.

*‘there were lots of people who wanted to do [TIVA] and on a day-to-day basis…one of the things was making it available and easy, so we had a Trust wide monitoring upgrade or replacement.’* (R, consultant anaesthetist)

Cost constraints were commonly cited. For example, survey respondents mentioned disincentives to using TIVA or RA owing to perceived expense or limited ultrasound machines.

*‘We are actively discouraged from using [TIVA] at my hospital due to the cost.’* (Consultant anaesthetist, survey)

Attempts to implement system changes were also hindered, with a surgeon reporting:

*‘Tried to develop a regional anaesthesia list… was told by management there’s no money.’* (Consultant surgeon, survey)

Efficiency and speed were often prioritised. Many surgeons perceived LA/RA as slower, whereas anaesthetists and nurses often viewed them as time-saving overall owing to quicker patient recovery and less need for anaesthetist oversight. In terms of TIVA, anaesthetists noted that TIVA can take slightly longer to administer than inhalation anaesthesia and may involve more labour or effort:

*‘I find TIVA slower…so that's why in my paediatric practice I actually still use quite a bit of volatile.’* (S, consultant anaesthetist)

Sustainability was not seen as a reason to change routine practice, with staff citing limited time and capacity in overstretched, under-resourced settings:

*‘environmental issues…are still pretty low down on people's agenda when you're working in an overrun, understaffed, underfunded hospital.’* (C, registrar surgeon)

Whereas a few participants viewed the environmental impact as an added benefit, most stated that their anaesthetic decisions were driven substantially by safety, efficiency, resource constraints, or institutional habits.

Nevertheless, some described a gradual shift toward sustainable practices, driven by improved outcomes and enhanced training. Several reported promoting LA/RA or TIVA within their departments:

*‘We've been talking about this for ages, you know, to do more hernias under local and stuff like that.’* (B, consultant surgeon)

Quantitative findings supported these themes. A majority of nurses (75; 68%) and anaesthetic assistants (34; 73%) reported faster operating times as a benefit of LA, but this was noted by only 13 (19%) surgeons and 18 (15%) anaesthetists. Across groups, 253 (73%) agreed that LA enables quicker patient discharge.

Sustainability ranked low as a factor in anaesthesia selection for both surgeons and anaesthetists. Most anaesthetists (82; 70%) reported that processed electroencephalography monitors for TIVA were always available, suggesting better access than some interviewees implied.

Surgeons agreed that scheduled sessions focused exclusively on procedures performed under LA are both feasible and efficient, whereas anaesthetists disagreed that time constraints lead them to prefer inhalation agents over TIVA (Table 6).

#### Theme 2—Patient differences and preferences

Patient comorbidities are a key factor in anaesthesia selection. As one anaesthetist noted:

*‘Comorbidities are the most likely thing to determining which anaesthetic is given.’* (S, consultant anaesthetist)

Other characteristics, such as anxiety, cognitive impairment, or communication barriers, were not contraindications but made LA/RA more challenging. These factors increased procedural complexity and stress for both staff and patients:

*‘patients with dementia…if you do them under regional, they'll be trying to climb off the bed…scared, and wondering what's going on.’* (Q, consultant anaesthetist)

Similarly, paediatric patients were commonly managed under GA:

*‘they're scared…and move around and they don't really obey commands, so we just put them to sleep.’* (D, senior house officer [SHO] surgeon)

Patient comfort was frequently raised, with participants describing the operating theatre as overwhelming, especially under LA. Patients may experience discomfort, including pain or sensations of ‘tugging and pulling’:

*‘it might be more traumatic…they can hear everything, they can see everything and sometimes…they actually feel some pain.’* (A, senior nurse)

Participants remarked that patient outcomes may vary depending on the type of anaesthesia used. Anaesthetists and surgeons noted that TIVA often yielded better outcomes than inhalation agents, such as less postoperative nausea and vomiting (PONV) and smoother recovery. LA/RA was seen as carrying fewer risks overall, although concerns remained about patient discomfort affecting surgical quality and patient outcomes:

*‘like if we do…under local and the patient can feel all of it, sometimes we don't clean it well enough…and then it could come back. So the chances of recurrence are higher under local.’* (E, SHO surgeon)

Patient preferences also influence decision making. Some patients prefer GA because of previous negative experiences or fear, whereas others welcomed the opportunity to remain awake and observe:

*‘Some people just don't want to know… whereas other people, they really want to watch.’* (R, consultant anaesthetist)

However, according to some participants, anaesthetic options are rarely discussed with patients in advance. Patients are often informed of the planned anaesthesia type by the surgeon, even when alternatives are available.

*‘Patients often come expecting GA as that’s what the surgeons told them or preop told them so it can be hard to alter that in their head on the day.’* (Consultant anaesthetist, survey)

Suggestions to address this included providing more information before surgery and using technology to reduce patient awareness during LA, for example, virtual reality (VR):

*‘technology and the use of VR and taking someone out of that world environment I think is a huge answer to that.’* (L, registrar surgeon)

In the survey, surgeons and anaesthetists rated patient preference and anxiety as leading situational factors influencing the selection of GA, with anxiety representing a clinically relevant but non-procedure-based consideration (Table 4). Both groups disagreed that patients tend to prefer LA when given a choice. In contrast to the qualitative findings, respondents generally agreed with the statement that they offer patients a choice between GA and LA/RA; however, when asked to rate agreement with the more general statement: ‘Patients are presented with a choice of local versus general anaesthesia’, mean ratings were substantially lower. Anaesthetists also strongly agreed that they use TIVA because it benefits patients (Table 6).

#### Theme 3—Influence of key decision makers on anaesthesia choice

Surgeons consistently reported a preference for GA:

*‘I think everybody really probably prefers it when the patient’s asleep.’* (C, registrar surgeon)

Reasons included ease of communication among surgeons, without the need to converse with the patient, avoidance of patient distress, and reduced self-consciousness among junior surgeons during training:

*‘…you want the patient to be asleep because you need to be able to ask…am I doing it correctly?…if I'm a bit nervous asking questions, the patient's not going to hear that, you know….’* (L, registrar surgeon)

By contrast, nurses and anaesthetists expressed fewer preferences. Some nurses welcomed patient interaction under LA, and anaesthetists appreciated the reduced involvement required:

*‘I’m a chatterbox and I don’t mind it.’* (J, ODP)

*‘I’d be happier to do it under local, because then I don't have to get involved.’* (Q, consultant anaesthetist)

Some participants became defensive when questioned further, citing past negative experiences with sustainability projects. One described eco-friendly gowns that leaked bodily fluids, resulting in a loss of trust:

*‘I wore the eco gowns and I was covered in urine and blood all the way down to my underwear because they ordered the wrong ones.’* (B, consultant surgeon)

Others questioned the focus on anaesthesia, suggesting environmental efforts should target more visible areas such as waste or energy use. A few remained unconvinced about TIVA’s environmental benefits:

*‘still not 100% convinced that TIVA is better than super low-flow volatile anaesthesia with sevoflurane to the environment.’* (Consultant anaesthetist, survey)

There was evidence of entrenchment: participants who initially acknowledged LA could improve outcomes sometimes reverted to defending GA, citing personal preferences or perceived patient expectations. Some argued that GA was insignificant compared with other environmental issues:

*‘what's the difference for a GA versus both the consultant and patient driving an hour to work in a diesel car…. There are so many other areas of our life which we could improve. Is focusing on GA really the answer?’* (C, registrar surgeon)

This pattern further highlights how environmental motivations, even when acknowledged, were often overridden by practical, cultural, or interpersonal factors, and patient safety and comfort.

Decisions were often shaped by professional hierarchies. Anaesthetists typically determined GA type (e.g. TIVA *vs* inhalation agents), whereas surgeons heavily influenced whether LA was used. For example, TIVA has increased where anaesthetists advocate for it, whereas surgeons’ preference for unconscious patients often limits LA use. This could facilitate or obstruct sustainable choices:

*‘if they [management] say we must do under local, it's very easy to produce a reason why you must not do it under local…surgeons would be the more vocal about it, cause at the end of the day, they're the ones doing the operation.’* (C, registrar surgeon)

Survey results supported these patterns. Anaesthetists strongly agreed that they decide the GA type. Across all groups, GA was perceived to reduce anxiety about patient pain, movement, and overheard conversations (see Table 5). Both surgeons and anaesthetists reported feeling more relaxed when patients were under GA, although most disagreed that they felt apprehensive performing procedures under LA/RA.

Despite scepticism, most participants supported making theatres more environmentally friendly, especially anaesthetists. Surgeons showed the highest agreement that reducing GA use could improve both patient care and sustainability. Notably, surgeons somewhat agreed and anaesthetists to a lesser extent agreed that LA/RA should be used where feasible. Both groups believed GA use could be reduced in their hospitals (Table 6). For general questions on beliefs and sustainability (i.e. ‘I believe the operating theatre needs to become more environmentally friendly’ and ‘to what extent would using LA where possible reduce the impact of operating theatres on the environment’), there was no difference in responses based on age or years of experience.

#### Theme 4—Default practices and lack of confidence in alternatives

GA remains the default anaesthetic approach in many settings:

*‘I think generally the culture is towards GA.’* (K, consultant surgeon)

Even when surgeons are happy to perform operations under LA, and have done so in the past, they default to GA, citing hospital culture as their reason. In contrast, for many anaesthetists, TIVA had become the norm:

*‘I’ve switched to it [TIVA] almost exclusively.’* (R, consultant anaesthetist)

One anaesthetist noted how default choices develop through repetition:

*‘In the theatre where I always use TIVA…I get in, I draw up my TIVA drugs and that's my standard practice. So my list that I've always traditionally used gas, I'm not in that mindset. I'm not drawing up the TIVA. …you do naturally do what you're used to doing.’* (S, consultant anaesthetist)

Some believed a cultural shift away from GA would not occur until generational change:

*‘until the new surgical era comes in…as surgeons we are super stubborn…it’s really difficult to change the culture.’* (O, consultant surgeon)

However, others changed behaviour even during the study. One consultant began offering LA more frequently after reflecting on their practice:

*‘nowadays after you started the project I basically started offering local for the opening lipomas much much more than before…. I found that more patients are keen to undergo the op under local anaesthetic than I was expecting…. It has helped a lot with reducing length of stay in elderly patients who were able to go home same day as they didn’t get GA… I am surprised that actually it works very well in the majority of the cases, patients’ feedback was great and nobody needed GA…’* (K, consultant surgeon)

Defaults were also shaped by local norms and resource availability. Participants described using LA abroad or at other NHS hospitals for the same procedures:

*‘I have no idea why it's not done under local. I have done it under local in this country.’* (E, SHO surgeon)

Training was another key factor. Limited exposure to LA/RA during surgical training contributed to lower confidence and increased anxiety:

*‘slightly more anxious, it’s another thing to consider, another thing to do…you're adding to the surgeon’s workload…you are still adding to the mental capacity, or the mental stress, of the operating surgeon… I haven't done that many under local to be fair.’* (C, registrar surgeon)

In contrast, growing training in TIVA has increased confidence among newer anaesthetists:

*‘the trainees that come through are doing so much more TIVA that for a lot of them their default is TIVA.’* (S, consultant anaesthetist)

Several participants emphasised that confidence builds with practice:

*‘recently I've done like local and sedation…it's going really well…kind of changed my mindset…because I was not very fond of the local because I didn't have much experience…some repetition and the trainees will become more familiar with the using of local anaesthetic.’* (K, consultant surgeon)

Survey data reflected these trends. Anaesthetists reported high confidence with spinal and sedation, but lower for other regional techniques. Surgeons expressed lower confidence operating under LA than RA or GA. Notably, both surgeons and anaesthetists agreed that some anaesthetists lacked confidence in RA. Nonetheless, most anaesthetists felt just as confident using TIVA as inhalation agents and disagreed that lack of TIVA training affected their decision. Over half reported using TIVA often or always.

## Discussion

This mixed-methods study examined barriers and facilitators to using LA/RA over GA, and TIVA instead of inhalation agents, in the UK. Key influences included time and efficiency pressures, patient experience, institutional defaults, and the influence of key decision makers. Although clinical factors appropriately guided decisions, environmental considerations were seldom incorporated when clinically suitable alternatives existed. However, many participants acknowledged that reducing GA use, where appropriate, could benefit both patients and the environment, aligning with wider calls for environmentally conscious perioperative practices.^38^, ^39^, ^40^

Time and efficiency dominated decision making. Surgeons tended to believe GA was quicker, despite nurses reporting shorter overall perioperative time with LA. These differing priorities align with research showing that surgeons commonly cite longer operating times as a key barrier to LA.^9^^,^^27^ Whereas LA/RA may reduce recovery time and waste, RA can increase total procedure duration in some cases, with implications for efficiency, cost, and environmental impact.^24^ These trade-offs highlight the importance of considering the full perioperative timeline and the context-specific impact of anaesthetic choices. For TIVA, the additional time required to prepare and administer drugs was cited as a barrier, consistent with existing literature.^38^, ^39^, ^40^

Several solutions were proposed to mitigate time-related barriers. Dedicated LA operating lists were seen as a way to reduce inefficiencies associated with case-by-case decisions. A previous study implementing LA-specific lists reported an 11-week reduction in waiting times, suggesting improved throughput.^41^ However, implementing and managing such lists require additional time and organisational support.^26^

Patient-related factors also strongly influenced anaesthetic choice. Whereas some patients prefer to remain awake and report high satisfaction with LA/RA,^41^ others experience anxiety or discomfort, key barriers to broader adoption. Surgeons noted that patient distress can affect surgical performance, for example, being more cautious when patients are awake or more forceful under GA, which may improve outcomes in some operations^27^ and reinforces patient preference as a major non-clinical driver. TIVA was widely seen as beneficial for patients, particularly in reducing PONV, consistent with earlier findings.^42^

To address patient anxiety, participants suggested better preoperative information and distraction technologies such as VR. Evidence supports the use of non-pharmacological interventions, including mobile applications^43^ and music,^44^ which can reduce preoperative anxiety, and serve as an effective alternative to sedation. Sedation itself, alongside LA, has also been shown to improve patients’ pain perception during surgery.^45^ In addition, patient involvement in anaesthesia decisions remained limited. Previous research found that although 71% of patients wished to participate in decision making, only 8% were invited to discuss their options.^46^

Institutional culture also shaped anaesthetic choice. Local norms, habits, and infrastructure often defaulted clinicians toward GA over LA/RA and inhalation agents over TIVA. Previous research shows that some anaesthetists prefer inhalation agents out of familiarity and comfort and are resistant to adopting TIVA.^26^ Participants noted that such defaults limit exposure to alternative approaches, reducing confidence, particularly in LA among surgeons and RA among some anaesthetists. Similar patterns have been reported elsewhere, where defaults contribute to limited LA expertise.^27^ These gaps in training and exposure highlight the need for targeted education and opportunities to practice lower-carbon techniques. However, defaults are not immutable: one surgeon in our study changed their practice during the study, offering more LA and reporting positive patient outcomes. This experience suggests that increased awareness and reflection on case-by-case choices may help reduce reliance on GA.^27^

The willingness of key decision makers to embrace change significantly influenced anaesthetic practice. Decisions were appropriately guided by patient outcomes; however, consistent with previous research,^26^ environmental factors were seldom considered alongside. Anaesthetists generally supported TIVA, often citing patient benefits, and were instrumental in its adoption; however, reluctance among some created substantial barriers.^26^ Although many surgeons endorsed LA in principle, they often deflected when asked why it was not used more in practice, echoing previous findings that clinicians may downplay their individual impact or emphasise broader systemic contributors to environmental harm.^26^

Although our findings offer useful insights, several limitations should be acknowledged. Interviews were conducted within a single NHS Trust, and although the survey was UK-wide, most responses were from England, particularly London, which may limit geographical representation. Recruitment materials referenced sustainability and may have attracted environmentally engaged participants, although survey data suggest this was not a primary driver of behaviour, reducing the likelihood of major sampling bias. Anaesthetic practice also varies internationally. For example, in some European countries, RA is used more routinely and by a broader range of clinicians,^47^ whereas in some low-and middle-income settings, limited anaesthetic workforce availability shapes how LA/RA techniques are delivered.^48^ In the USA and UK, LA is often performed without an anaesthetic team. Such contextual differences should be considered when interpreting transferability. Finally, this study focused on two major anaesthetic decisions with known environmental impact—the choice between GA and LA/RA, and between TIVA and inhalation agents. Other sustainability-related practices, including oxygen flow rates, equipment selection, and perioperative behaviours, were beyond scope and are areas for future research.

In conclusion, anaesthetic decision making is clinically driven but shaped by time pressures, patient concerns, default practices, and the influence of key decision makers. Although sustainability is rarely a primary driver where clinical choices exist, shifts motivated by patient outcomes, such as the increased adoption of TIVA to reduce PONV, demonstrate that change is possible when clinical and environmental benefits align. Promoting more sustainable, patient-centred practices will require addressing both systemic and behavioural barriers. Future work should focus on co-designing behaviourally informed interventions with surgeons, anaesthetists, and patients to ensure clinical feasibility, acceptability, and long-term sustainability.

## Authors' contributions

Conception and design of this research: all authors

Acquired and analysed the data: CB, AA, AM

Interpreted the data: all authors

Drafted the manuscript: CB

Revised the manuscript critically for important intellectual content: all authors

Read and approved the final manuscript: all authors

## Data availability statement

The datasets used, analysed, or both during the current study are available from the corresponding author on reasonable request.

## Funding

Medical Research Council (grants MR/X011720/1 and MR/X502959/1); National Institute for Health and Care Research.

## Declaration of interest

The authors declare that they have no conflicts of interest.
