# Supplementary material for: Barriers and enablers to sustainable anaesthetic practice: a mixed-methods study
Source: Br J Anaesth. 2025 Dec 17;136(4):1190–201. doi: 10.1016/j.bja.2025.11.009 (PMC13014499; doi:10.1016/j.bja.2025.11.009)
Supplement: Multimedia component [file mmc1.docx]

### Appendix 1 – Interview Topic Guide


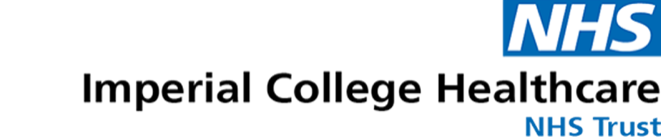


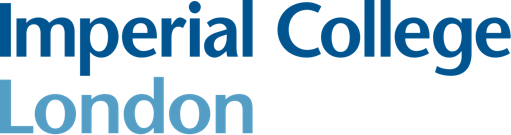


**Topic Guide**

**A qualitative study to engage key stakeholders to identify the facilitators and barriers to the adoption of green surgery practices and co-design interventions**

**Introduction**

Thank you for taking the time to talk with me today.

The aim of this interview is to understand more about barriers and facilitators to reducing the environmental impact of operating theatres in the UK.

Please feel free to skip over any questions you do not wish to answer, and to answer in as much or as little detail as you would like. Your responses will remain anonymous. Let me know if you would like to take a break or stop the interview at any point.

Do you have any questions?

I’ll start recording now, is that okay?

*Start recording and transcribing*

*Ask everyone opening questions in part 1, in part 2 ask the appropriate questions for that role, and ask everyone part 3 closing questions*

**PART 1: OPENING QUESTIONS**

**Can you start by telling me a little bit about yourself. What is your current professional role?**

**Which operations do you perform or participate in?**

E.g. which specialties, which types of operations, duration etc

**PART 2a: NURSES**

**Thank you. We’ll now focus on the use of regional / local anaesthesia and general anaesthesia.**

**For surgeries where there is the choice between local/regional or general anaesthetic, do you have a preference for working with one type or the other?**

Does the choice of anaesthetic affect your role? In what ways?

In which cases would you prefer a regional/local or general anaesthetic?

**PART 2b: ANAESTHETISTS.**

**Thank you. We’ll now focus on the use of regional / local anaesthesia and general anaesthesia.**

| **Question** | **TDF** |
| --- | --- |
| **We know there are some surgeries, e.g. inguinal hernia repair, and lipoma removal that can be done under local or general. We are interested in why you may choose one type of anaesthetic rather than another. In general, regardless of the operation, do you prefer to anaesthetise under general or regional anaesthesia? Why?** | Knowledge |
| Whose role or responsibility is it to adhere to these guidelines? Why? Whose responsibility do you think it should be? |  |
| **Do you think the hospital/management support efforts to reduce the number of surgeries performed using general anaesthesia?** | Social/ professional role and identity |
| In what way? Or - Why not? How could it be better? |  |
| Do you think surgeons would support? Do you think patients support? Why/why not? |  |
| **Would reducing the number of surgeries using general anaesthesia fit into your workplace culture?** | Social influences |
| Would your team members in the operating theatre support this? |  |
| What you know of specialties where there is a push for the use of regional/local anaesthesia? Can we learn something from them? |  |
| Do surgeons have a different opinion or preference on this compared to anaesthetists? |  |
| Does this differ based on the type of operation? - thinking about operations which can be done either way (give examples from earlier) |  |
| Do you feel this influence from colleagues affects your choice? For example, senior anaesthetists or surgeons? |  |
| Are there team members who would encourage using general anaesthesia, even when unnecessary? Why? |  |
| Have you been in teams where it would be difficult to advocate for using less general anaesthesia? |  |
| **What is it about the operating theatre environment that facilitates or challenges your ability to use local or regional anaesthesia?** | Environmental context and resources |
| Is equipment for general anaesthesia more readily available? |  |
| Are there other non-clinical reasons why it may be easier or preferred to use one rather than the other? |  |
| **Do you feel capable of increasing your use of regional anaesthesia?** | Beliefs about capabilities |
| If not, what makes it harder for you to do this? |  |
| What are the factors that would influence this? Do they change depending on the operation? |  |
| **If you think about using more local and less general anaesthesia in surgeries, how does that make you feel?** | Emotion |
| E.g. anxious, motivated. Why? |  |
| **Do you have any goals for reducing use of general anaesthesia?** | Memory, attention, decision |
| Do you have different goals for different surgeries? |  |
| **Do you know of any incentives for reducing general anaesthesia in operating theatres?** | Reinforcement |
| **Do you think it is likely that the use of general anaesthesia can be reduced in your hospital?** | Optimism |
| Why/why not? |  |
| **Do you think it is likely that you will reduce your use of general anaesthesia?** | Optimism |
| Why/why not? |  |

**We now have some questions about TIVA**

| **Question** | **TDF** |
| --- | --- |
| **Who decides which type of general anaesthesia to use (volatile vs TIVA)** | Decision making |
| **Do you tend to use TIVA?**  Does this differ on the case?  In what way? | Behaviour |
| **Do you consider the environmental impact when selecting the type of general anaesthesia?** | Decision making |
| **Do you feel just as confident using TIVA as you do using gases for general anaesthesia?** | Beliefs about capabilities |
| **Were you trained in using TIVA?**  By who? | Skills |
| **Does your hospital use volatile gas capture technology?** | Context and resources |
| **Are there Depth of Anaesthesia pEEG monitors (eg BIS, Entropy) readily available for use in the operating theatres you work in?** | Context and resources |
| **Is TIVA encouraged for general anaesthetic in your hospital?** | Social influences |
| **What are the benefits of TIVA against gases?**  for the patient, the environment, cost, time taken for patient and total turnaround time | Context and resources |
| **Do you have any further comments on using TIVA compared to gases for general anaesthesia?** | **-** |

**PART 2c: SURGEONS**

**Thank you. We’ll now focus on the use of regional / local anaesthesia and general anaesthesia. This list is designed to gain insights from all grades. Some might apply less to you than a junior/senior surgeon.**

| **Question** | **TDF** |
| --- | --- |
| **We know there are some surgeries, e.g. inguinal hernia repair, and lipoma removal that can be done under local or general. We are interested in why you may choose one type of anaesthetic rather than another. In general, regardless of the operation, do you prefer to operate under general or regional/local anaesthesia? Why?**  **In your speciality, which are the surgeries where the surgical outcome is not affected by type of anaesthesia?**  **Do you decide which type of anaesthesia to use? When is this decision made? Do you discuss with the anaesthetist and patient? When? Can this decision change e.g. on the day? Why? How much influence does the anaesthetist have?**  **Would you factor in anaesthetist confidence in using regional anaesthetic?** | Knowledge |
| If there is no clinical contraindications to using regional/local anaesthesia, what other factors do you take into account when deciding on anaesthesia type?  Do you think there is a scope to do more operations under regional anaesthesia that are usually done under local? E.g. Coloproctology operations under spinal or epidural? |  |
| Who decides? Who has more influence of the decision? Is the operating surgeon or the anaesthetist? |  |
| When is the final decision made? |  |
| When do you talk to the patient about type of anaesthesia?  In your experience, do patients prefer regional/local or general? |  |
| **Do you think there is a need to reduce the number of surgeries using general anaesthetic in your hospital?**  Do you support moving to regional? Why/why not? | Intentions |
| Do you think efforts should be made to reduce this? |  |
| Can you elaborate? |  |
| Had you thought before about the environmental impact of using general anaesthesia instead of local? In what way? |  |
| **If the number of surgeries using general anaesthesia was reduced, what impact do you think this would have on the environment?** | Beliefs about consequences |
| **And on patient care?** |  |
| **Would the set of skills needed to perform an operation change when doing the same surgery under regional/ local or general?** | Skills |
| Do you have similar amount of experience with each type? Are you just as confident with using local anaesthesia? |  |
| For operations which are usually done under general anaesthesia, would be just as confident doing them under regional/local? |  |
| **Do you know of recommendations or guidelines for reducing the use of general anaesthesia in surgery?** | Knowledge |
| If yes - |  |
| What do these guidelines recommend? |  |
| Where do you find/get this knowledge? (is it from hospital/trust/elsewhere?) |  |
| How readily available is this information? |  |
| Are there any challenges to accessing this information? |  |
| Do many people know about this? |  |
| Whose role/responsibility is it to adhere to these guidelines? Why? Whose responsibility should it be? |  |
| **Do you think the hospital/management support efforts to reduce the number of surgeries performed using general anaesthesia?** | Social/ professional role and identity |
| In what way? Or - Why not? How could it be better? |  |
| Do you think anaesthesia would support? Do you think patients support? Why/why not? |  |
| **Would reducing the number of surgeries using general anaesthesia fit into your workplace culture?** | Social influences |
| Would your team members in the operating theatre support this? |  |
| What you know of specialties where there is a push for the use of regional/local anaesthesia?  Can we learn something from them? |  |
| Do surgeons have a different opinion or preference on this compared to anaesthetists? |  |
| Does this differ based on the type of operation? - thinking about operations which can be done either way (give examples from earlier) |  |
| Do you feel this influence from colleagues affects your choice? For example senior anaesthetists or senior surgeons? |  |
| Are there team members who would encourage using general anaesthesia, even when unnecessary? Why? |  |
| Have you been in teams where it would be difficult to advocate for using less general anaesthesia? |  |
| **What is it about the operating theatre environment that facilitates or challenges your ability to use local or regional anaesthesia?** | Environmental context and resources |
| Is equipment for general anaesthesia more readily available? |  |
| Are there other non-clinical reasons why it may be easier or preferred to use one rather than the other? |  |
| **Do you feel capable of increasing your use of local/regional anaesthesia?** | Beliefs about capabilities |
| If not, what makes it harder for you to do this? What are the factors that would influence this? Do they change depending on the operation? |  |
| **If you think about using more local/regional and less general anaesthesia in surgeries, how does that make you feel?** | Emotion |
| E.g. anxious, motivated. Why? |  |
| **Do you have any goals for reducing use of general anaesthesia?** | Memory, attention, decision |
| Do you have different goals for different surgeries? |  |
| **Do you know of any incentives for reducing general anaesthesia in operating theatres?** | Reinforcement |
| **Do you think it is likely that the use of general anaesthesia can be reduced in your hospital?** | Optimism |
| Why/why not? |  |
| **Do you think it is likely that you will reduce your use of general anaesthesia?** | Optimism |
| Why/why not? |  |

**PART 3: CLOSING QUESTIONS**

**Do you anticipate any further barriers to trying to change these behaviours in the operating theatre?**

**Are there any other factors that help you to use local anaesthetic not general?**

Something that already exists e.g. a poster or other campaign, or a particular colleague, or your behaviour/values outside of work, RCS checklist

Do other campaigns e.g. hand hygiene within the Trust or a reduction in desflurane (pick suitable example) influence your behaviour in the operating theatre/in this area?

**Do you have any ideas about how the implementation of measures to reduce the environmental impact of operating theatres can be improved? Is there one thing that would make it easier or motivate you and others to do so?**

Some ideas are: Support or feedback from colleagues; education around impact; incentives

**And finally, this has been about the operating theatre. How might this relate to reducing your impact in other parts of your clinical role?**

E.g. for nurses - Are there different things that make it easier or harder to reduce unnecessary glove use outside of theatre compared to in theatre?

**This is one stage of the project, but we are hoping to look at other areas of the operating theatre in future stages - do you have any other areas you think are important?**

### Appendix 2 - All survey questions

| **TDF/ other domain** | **Survey item** | **Response options** | **Nurses** | **Surgeons** | **Anaesthetists** |  |
| --- | --- | --- | --- | --- | --- | --- |
| **LOCAL VS GENERAL ANAESTHESIA** | | | | | |  |
| Behaviour | Almost all my operations are performed under general anaesthesia | Tick appropriate response |  | X |  |  |
|  | Almost all of my operations are performed under local, including regional or spinal anaesthesia |  |  |  |  |  |
|  | Some of my operations are performed under general anaesthesia and some under local or regional/spinal anaesthesia |  |  |  |  |  |
|  | Are there any operations that you currently perform under general anaesthesia which could be performed under local or regional anaesthesia? (i.e. do you know that some other institutions or surgeons perform them under local or regional anaesthesia?) | Yes, no, or not sure |  | X |  |  |
| Memory, attention and decision processes | Do you have a preference to work in an operation under general anaesthesia or local? | I prefer local, I prefer general, or no preference | X | X |  |  |
| Memory, attention and decision processes | The surgery takes less time | Tick to indicate preferred aspects of local anaesthesia | X | X | X |  |
|  | Less patient monitoring required |  |  |  |  |  |
| Social influences | I can talk to the patient |  |  |  |  |  |
| Beliefs about capabilities | The surgery is easier (relative to general anaesthesia) |  |  |  |  |  |
| Beliefs about consequences | The patient can leave shortly after the surgery |  |  |  |  |  |
|  | It is safer for the patient |  |  |  |  |  |
|  | It's better for the environment |  |  |  |  |  |
| Social influences | No concerns about communicating with the trainer or trainee | Tick to indicate preferred aspects of general anaesthesia | X | X | X |  |
|  | No concerns about discussions within the team that would be inappropriate or distressing for the patient to hear |  |  |  |  |  |
| Beliefs about capabilities | The surgery is easier (relative to local/regional anaesthesia) |  |  |  |  |  |
| Emotion | I don’t have to worry about the patient feeling pain |  |  |  |  |  |
|  | No concerns about the patient feeling anxious |  |  |  |  |  |
| Memory, attention and decision processes | I don't have to worry about the patient moving during the operation |  |  |  |  |  |
| Environmental context and resources | The surgery takes less time |  |  |  |  |  |
| Environmental context and resources | If there is no clinical contraindication to using regional/local anaesthesia, how often do these factors lead you to select general rather than local/regional? Likelihood of patient movement | 1 (never) to 5 (always) |  | X | X |  |
|  | Patient preference |  |  |  |  |  |
|  | Language translation of communication to patient if they are awake |  |  |  |  |  |
|  | Anxiety levels of patient |  |  |  |  |  |
|  | Time taken to perform surgery |  |  |  |  |  |
|  | The number of surgeries you have that day |  |  |  |  |  |
|  | Time of day of the operation |  |  |  |  |  |
|  | Confidence with administering regional block required |  |  |  |  |  |
| Memory, attention and decision processes | I always present the patient with a regional or local anaesthetic option as well as a GA option when clinically appropriate (not including supplementary regional anaesthesia) | 1 (disagree) to 5 (agree) |  |  |  |  |
|  | When deciding on which anaesthesia to use (general, local or regional), I consider the environmental impact |  |  | X | X |  |
|  | I prefer to perform operations under general anaesthesia instead of local anaesthesia/I prefer to use general anaesthesia versus regional anaesthesia |  |  | X | X |  |
|  | Having a dedicated LA/regional operating list is feasible and efficient |  |  | X |  |  |
| Beliefs about capabilities | I feel just as confident using local anaesthesia only as I do in a surgery where the patient is under general anaesthesia | 1 (disagree) to 5 (agree) |  | X |  |  |
|  | I feel confident providing epidural/spinal anaesthesia |  |  |  | X |  |
|  | I feel confident providing other regional anaesthesia as needed for my practice |  |  |  | X |  |
|  | I feel confident providing sedation to a patient under local anaesthesia |  |  |  | X |  |
| Environmental context and resources | It takes me longer to anaesthetise using regional anaesthesia than it does general | 1 (disagree) to 5 (agree) |  |  | X |  |
|  | Operations under local anaesthesia take me longer than operations under general anaesthesia |  |  | X |  |  |
|  | Some anaesthetists I work with are not as confident doing regional anaesthesia as they are doing general |  |  | X | X |  |
|  | Using general anaesthesia is the default option |  |  | X | X |  |
| Goals | We should always aim to do operations under local/regional anaesthesia, if possible | 1 (disagree) to 5 (agree) |  | X | X |  |
| Emotion | I feel more relaxed in an operation which is performed under general anaesthesia/I feel more relaxed anaesthetising using general anaesthesia | 1 (disagree) to 5 (agree) |  | X | X |  |
|  | I feel apprehensive when I have to perform an operation using local anaesthesia /I feel apprehensive when anaesthetising using regional anaesthesia |  |  |  |  |  |
| Beliefs about consequences | If the number of operations using general anaesthesia was reduced, it would have a positive impact on.. the environment | 1 (disagree) to 5 (agree) |  | X | X |  |
|  | patient care |  |  |  |  |  |
|  | To what extent would using local anaesthetic where possible, instead of general reduce the impact of operating theatres on the environment? | 1 (minimal impact on the environment) to 5 (large impact on the environment) | X | X | X |  |
| Social influences | Patients are presented with a choice of local versus general anaesthesia (when possible) | 1 (disagree) to 5 (agree) |  | X | X |  |
|  | My patients tend to prefer general anaesthesia if given the choice |  |  |  |  |  |
|  | My patients tend to prefer local anaesthesia if given the choice |  |  |  |  |  |
|  | The surgical team I am working with is open to using local/regional anaesthesia instead of general for an operation which could use either |  |  |  |  |  |
| Optimism | It is likely that the use of general anaesthesia can be reduced in my hospital | 1 (disagree) to 5 (agree) |  | X | X |  |
|  | The hospital I work in would support efforts to reduce the number of operations done under general anaesthesia |  |  |  |  |  |
| Social influences | How competent do you believe your NHS Trust is in… protecting you from infections? | 1 (not at all competent) to 5 (very competent) | X | X | X |  |
|  | protecting patients from infections? |  |  |  |  |  |
|  | How committed do you believe your NHS Trust is to… protecting you from infections? | 1 (not at all committed) to 5 (very committed) | X | X | X |  |
|  | protecting patients from infections? |  |  |  |  |  |
| Goals | I believe the operating theatre needs to become more environmentally friendly | 1 (disagree) to 5 (agree) | X | X | X |  |
| / | Do you have any other comments about influences on doing more surgeries under local/regional instead of general (when clinically possible)? | - | X | X | X |  |
| **TOTAL INTRAVENOUS ANAESTHESIA** | | | | | |  |
| Behaviour | When providing a general anaesthetic, I use TIVA rather than gases | 1 (never) to 5 (always) |  |  | X |  |
| Memory, attention and decision processes | It is my decision which type of general anaesthesia to use (volatile vs TIVA) | 1 (disagree) to 5 (agree) |  |  | X |  |
| Behavioural regulation | I consider the environmental impact when selecting the type of general anaesthesia | 1 (disagree) to 5 (agree) |  |  | X |  |
| Beliefs about capabilities | I feel just as confident using TIVA as I do using gases for general anaesthesia | 1 (disagree) to 5 (agree) |  |  | X |  |
| Environmental context and resources | My hospital uses volatile gas capture technology | 1 (yes, in all theatres), 2 (yes, in most theatres), 3 (yes, in some theatres), 4 (no), 5 (I don't know) |  |  | X |  |
|  | There are Depth of Anaesthesia pEEG monitors (eg BIS, Entropy) readily available for use in the operating theatres I work in | 1 (never) to 5 (always) |  |  | X |  |
|  | I decide to use gases rather than TIVA because TIVA takes longer | 1 (disagree) to 5 (agree) |  |  | X |  |
| Social influences | TIVA is encouraged for general anaesthetic in my hospital | 1 (disagree) to 5 (agree) |  |  | X |  |
| Beliefs about consequences | I believe TIVA is better for the environment (than non-captured volatile gases) | 1 (disagree) to 5 (agree) |  |  | X |  |
|  | I decide to use TIVA as it's better for the patient |  |  |  |  |  |
| Skills | I decide to use gases as I am not trained in using TIVA | 1 (disagree) to 5 (agree) |  |  | X |  |
| / | Do you have any comments on using TIVA compared to gases for general anaesthesia? | - |  |  | X |  |
